# Tin and Oxygen-Vacancy Co-doping into Hematite Photoanode for Improved Photoelectrochemical Performances

**DOI:** 10.1186/s11671-020-3287-1

**Published:** 2020-03-04

**Authors:** Chenhong Xiao, Zhongyuan Zhou, Liujing Li, Shaolong Wu, Xiaofeng Li

**Affiliations:** 1 School of Optoelectronic Science and Engineering & Collaborative Innovation Center of Suzhou Nano Science and Technology, Soochow University, Suzhou, 215006 Jiangsu China; 2 Key Laboratory of Advanced Optical Manufacturing Technologies of Jiangsu Province & Key Laboratory of Modern Optical Technologies of Education Ministry of China, Soochow University, Suzhou, 215006 Jiangsu China

**Keywords:** Hematite, Photoelectrochemical water splitting, Ultrasonic spray pyrolysis, Sn doping, Oxygen vacancy

## Abstract

Hematite (α-Fe_2_O_3_) material is regarded as a promising candidate for solar-driven water splitting because of the low cost, chemical stability, and appropriate bandgap; however, the corresponding system performances are limited by the poor electrical conductivity, short diffusion length of minority carrier, and sluggish oxygen evolution reaction. Here, we introduce the in situ Sn doping into the nanoworm-like α-Fe_2_O_3_ film with ultrasonic spray pyrolysis method. We show that the current density at 1.23 V vs. RHE (*J*_ph@1.23V_) under one-sun illumination can be improved from 10 to 130 μA/cm^2^ after optimizing the Sn dopant density. Moreover, *J*_ph@1.23V_ can be further enhanced 25-folds compared to the untreated counterpart via the post-rapid thermal process (RTP), which is used to introduce the defect doping of oxygen vacancy. Photoelectrochemical impedance spectrum and Mott-Schottky analysis indicate that the performance improvement can be ascribed to the increased carrier density and the decreased resistances for the charge trapping on the surface states and the surface charge transferring into the electrolyte. X-ray photoelectron spectrum and X-ray diffraction confirm the existence of Sn and oxygen vacancy, and the potential influences of varying levels of Sn doping and oxygen vacancy are discussed. Our work points out one universal approach to efficiently improve the photoelectrochemical performances of the metal oxide semiconductors.

## Background

The conversion of solar energy into hydrogen by photoelectrochemical (PEC) water splitting has been a major research direction for scientists in new energy [1–4]. In the related investigations, the exploitation of the photoactive material is crucial. For now, numerous metal oxides (e.g., WO_3_ [5], BiVO_4_ [6], and TiO_2_ [7]) have been constructed into photoanodes for water oxidation due to the excellent chemical stability and the maximum of valence band positive to the potential of H_2_O/O_2_. Among them, hematite (α-Fe_2_O_3_) is particularly promising benefited from the suitable bandgap (~ 2.1 eV) for the absorption of visible light, vast abundance of the consisting elements, non-toxicity, low-cost preparation, and so on [8–10]. However, its practical efficiency is far less than the theoretical limit mainly due to the poor conductivity, short hole diffusion length, and slow hole kinetics [11–13].

The methods for resolving these problems include element doping (to improve the photoactive-material conductivity) and surface modification of the photoelectrode surface (to enhance the surface reaction kinetics or to suppress the surface carrier recombination) [14–17]. Doping of α-Fe_2_O_3_ with moderate additives such as Zr^4+^ [18], Ti^4+^ [8], Sn^4+^ [19], and Al^3+^ [20] can improve the conductivity and then reduce the obstruction of the carrier collection. Moreover, the short hole diffusion length makes it difficult for the extraction of the photogenerated holes to the photoanode surface for water oxidation. It is thus significant to take some methods to control the morphology of hematite film. For instance, Sivula et al. reported a mesoporous hematite with improved PEC performance after using 800 °C annealing, and deemed that the performance enhancement is due to the Sn diffusion from the FTO substrate during the annealing treatment [21]. Ling et al. further revealed that the internal mechanism of Sn doping could be achieved at a relatively low temperature (i.e., 650 °C) [22]. However, most reports used diffusion or non-quantitative method to introduce additive elements because few preparation technologies could quantitatively introduce dopant in spite of many methods being developed for growing α-Fe_2_O_3_, such as atomic layer deposition (ALD) [23], atmospheric pressure chemical vapor deposition (APCVD) [24], electro-chemical deposition [25], pyrolysis [26], and hydrothermal methods [27]. Non-quantitative analysis cannot exactly discover the change in crystallinity and composition as the doping density changes. In general, too-low level of doping cannot adequately increase the conductivity, while too-high level results in the reduced efficiency of the photogenerated carrier due to the increased bulk recombination.

The concept of oxygen vacancy started in 1960s [28]. In the beginning, oxygen vacancy was used to study the gas contacted with surface of metal. Then, it was found that it could be used as active site to improve the PEC performance. Oxygen vacancy appears in the special conditions like rapid thermal process (RTP) [28], which can bring about oxygen separation from the metal-oxidation lattice [29]. Currently, oxygen vacancies are regarded as intrinsic defects and positive charges because of the strong electronegativity of oxygen. For the defect-doped α-Fe_2_O_3_ photoanode, the understanding of oxygen vacancy is not comprehensive.

In this study, we use ultrasonic spraying to grow α-Fe_2_O_3_ film. By controlling the molar ratio of Sn^4+^ and Fe^3+^ in the precursor solution, α-Fe_2_O_3_ with a relatively accurate Sn doping can be achieved. We observed that the *J*_ph@1.23V_ of α-Fe_2_O_3_ photoanode with optimal Sn doping under one-sun illumination could be enhanced 13-folds relative to the 0% doped situation (i.e., the case without Sn element in precursor solution), and it can be further improved to 25-folds after the optimized post-RTP treatment. The primary influences of Sn doping and RTP are analyzed from different perspectives. We believe that this work provides a new possibility to introduce performance-improved methods for the different metal oxide semiconductors in the field of solar energy conversion.

## Methods

### Materials

Ferric nitrate [Fe (NO_3_)_3_, 98.5 wt%] and tin tetrachloride pentahydrate [Sn (Cl)_4_, 98 wt.%] are supplied by Aladdin Regent Company. Acetone, ethyl alcohol, and sodium hydroxide (NaOH) are purchased from Sinopharm Chemical Reagent Co., Ltd. All water used in the process of the experiment is deionized water (18.25 MΩ•cm). The conductive substrate is the fluorine-doped tin oxide (FTO, 7 Ω sq.^−1^) glass. All reagents and materials are of analytical grade without any purification.

### Hematite Preparation

The α-Fe_2_O_3_ is synthesized on the FTO substrate by ultrasonic spraying (HZAC200, Hizenith Robots Co., Ltd.). The detailed processes are as following: (1) FTO substrates are cleaned respectively with acetone, ethyl alcohol, and deionized water for 15 min by ultrasonic; (2) changing the hydrotropism of FTO substrate by oxygen plasma cleaning; (3) preparing the precursor solution should be exquisite. First, certain amounts of Fe (NO_3_)_3_ and Sn (Cl)_4_ are dissolved in ethanol. Second, stir the mixed solution for 10 min to make sure that there is no macroscopic insoluble material. Third, the ultrasonic treatment of mix solution is used to eliminate the bubbles that could result in the drop in the spraying process; (4) 5 mM Fe (NO_3_)_3_ ethanol solution is sprayed on the FTO for 30 min (from Figure S1, spraying for 30 min corresponds to the most suitable thickness of around 120 nm). Note that the FTO substrates are fixed on the heating plate with a distance of ~ 11 cm from the sprayer, and the surface of the sample holder is fixed to a constant temperature of 80 °C, along with a constant rotational speed of 100 r/min. The thickness of α-Fe_2_O_3_ is determined by the spraying time; (5) after spraying, the as-deposited thin film is heated at 700 °C for 2 h and then the Sn-doped α-Fe_2_O_3_ film is obtained; (6) finally, RTP (RTP500, Beijing East Star Co., Ltd.) is carried out under nitrogen atmosphere for 90 s with varying temperatures.

### Characterizations of Structure and Material

The sample morphology is examined through field emission scanning electron microscopy (SEM Hitachi S4800). Transmission electron microscopy (TEM, FEI Tecnai G2 F20 S-Twin) is used to perform the subtle information of structure. The crystal phase is confirmed by X-ray diffraction (XRD, MRD X’Pert-Pro) equipped with Cu kα radiation. The details of composition about hematite are provided by Raman spectroscopy (HR800 LabRAM) and X-ray photoelectron spectroscopy (XPS, ESCALAB 250Xi). The absorption spectrum is obtained by unity subtracting the transmission and reflectance, which are measured by a spectrophotometer (HORIBA, iHR320) equipped with an integrating sphere and commercial detectors.

### PEC Performance Measurements

The as-prepared α-Fe_2_O_3_ film on FTO is made into photoanode. To start with, we coat In/Ga film on the conductive part of FTO glass and then stick the Cu wire to the In/Ga part of FTO substrate with hot melt adhesive. In addition, the silica gel is used to cover the connection part between Cu wire and α-Fe_2_O_3_ film grown on the FTO glass to control the portion directly contacted to the electrolyte (i.e., 1 M NaOH aqueous solution). Finally, potential is applied relative to the Ag/AgCl reference electrode. Current density vs potential (*J-V*) curves are obtained by scanning potential ranging from − 1 to 0.7 V (relative to the reference electrode) at a scan rate of 20 mV/s under one-sun simulator (SS-F7-3A, Enlitech). The applied potential vs Ag/AgCl is converted to potential vs RHE by Nernst equation:

1 $$ {E}_{\mathrm{RHE}}={E}_{\left(\mathrm{Ag}/\mathrm{AgCl}\right)}+0.059\times \mathrm{pH}+{E}_{\left(\mathrm{Ag}/\mathrm{AgCl}\right)}^0 $$

where *E*_RHE_ is the converted potential vs RHE, *E*^0^_Ag/AgCl_ = 0.1976 V at 25 °C, and *E*_Ag/AgCl_ is the experimentally employed potential against Ag/AgCl reference. The as-prepared photoanodes are used as working electrode to carry out PEC test in the electrochemical workstation (CIMPS, Zennium Zahner).

Electrochemical impendence spectrum (EIS) is obtained in 1 M NaOH electrolyte at a bias of 0.23 V vs Ag/AgCl under the one-sun illumination over the frequency of 100 kHz to 0.1 Hz. Mott-Schottky plots are measured in the same electrolyte at the frequency of 1 kHz at a bias ranging from − 1 to 0.7 V (relative to the Ag/AgCl). Incident photon-to-current efficiency (IPCE) spectrum is conducted at a bias of 0.23 V vs Ag/AgCl under varying wavelengths from 300 to 700 nm.

## Results and Discussion

**Fig. 1:**
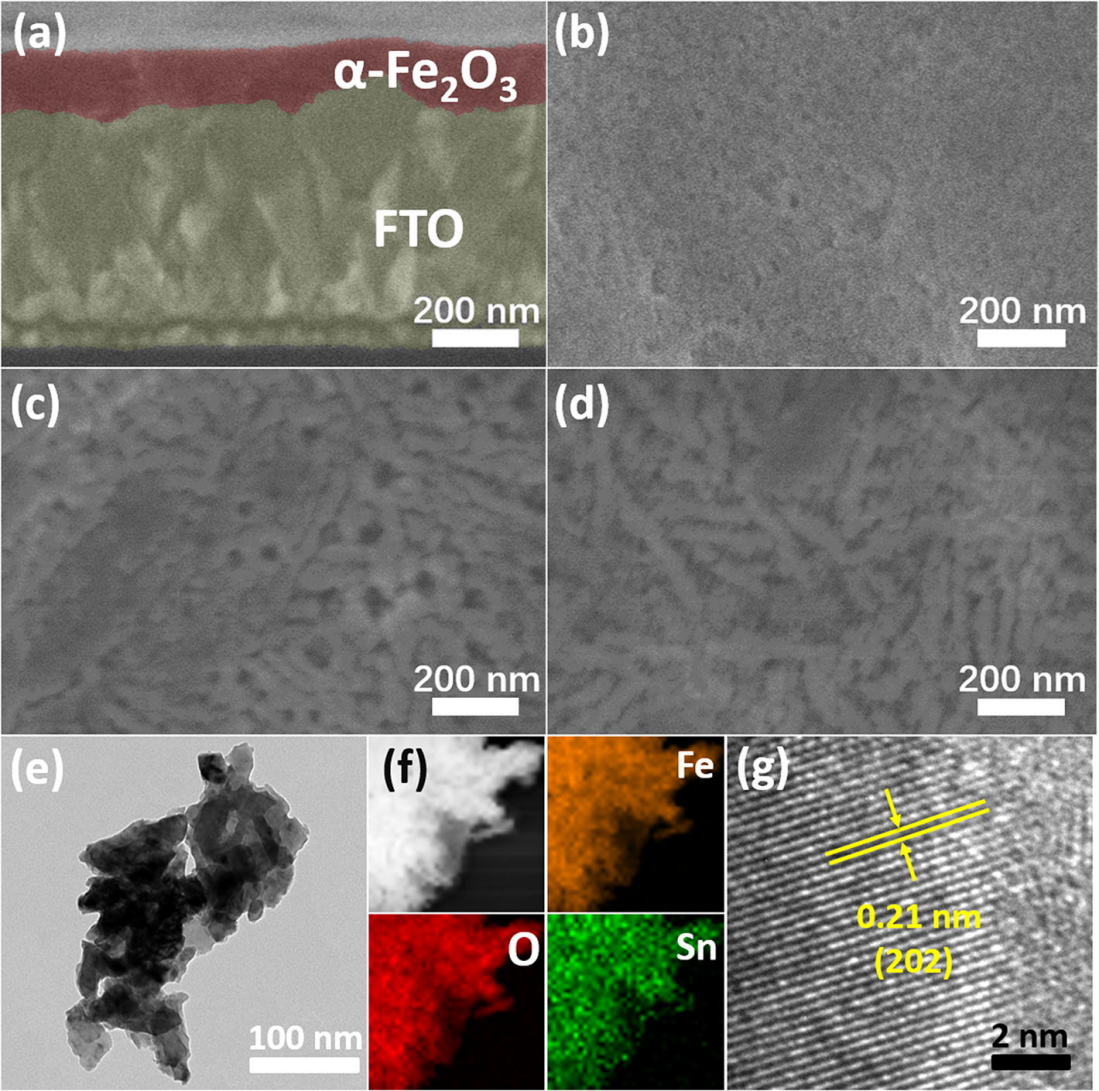
Representative SEM and TEM photos of the synthesized α-Fe_2_O_3_ film. **a**, **b** The cross-sectional and top-view SEM images of the sample with 0% doping and no-RTP. **c** The top-view SEM image of the 15% Sn-doped sample. **d** The top-view image of the 15% Sn-doped hematite with post-RTP. **e** TEM image of Sn-doped hematite with post-RTP. **f** STEM image of partial region in **e**. **g** HRTEM image of the Sn-doped α-Fe_2_O_3_ with post-RTP

To figure out the relationship between the growth methods and PEC responses of α-Fe_2_O_3_ photoanode, one should first focus on the morphology. Figure 1a shows that compact and uniform α-Fe_2_O_3_ film with a thickness of ~ 120 nm is grown on FTO substrate. Compared with Fig. 1b, one can see the great influence on the morphology from Sn doping (Fig. 1c). The interval distance between neighbor α-Fe_2_O_3_ nanoparticles becomes larger after introducing the external Sn doping, and the shape of nanoparticles changes to the nanoworm-like. In Fig. 1d, the nanoparticles become thinner and longer relative to those in Fig. 1c. The mixture of many nanoworm-like α-Fe_2_O_3_ particles is shown in Fig. 1e. With the STEM and the corresponding elemental mappings, one can see that Fe, Sn, and O elements are uniformly distributed in the prepared nanoparticles (Fig. 1f). High-resolution TEM (HRTEM) images reveal the lattice structure of α-Fe_2_O_3_ (Fig. 1g).

**Fig. 2:**
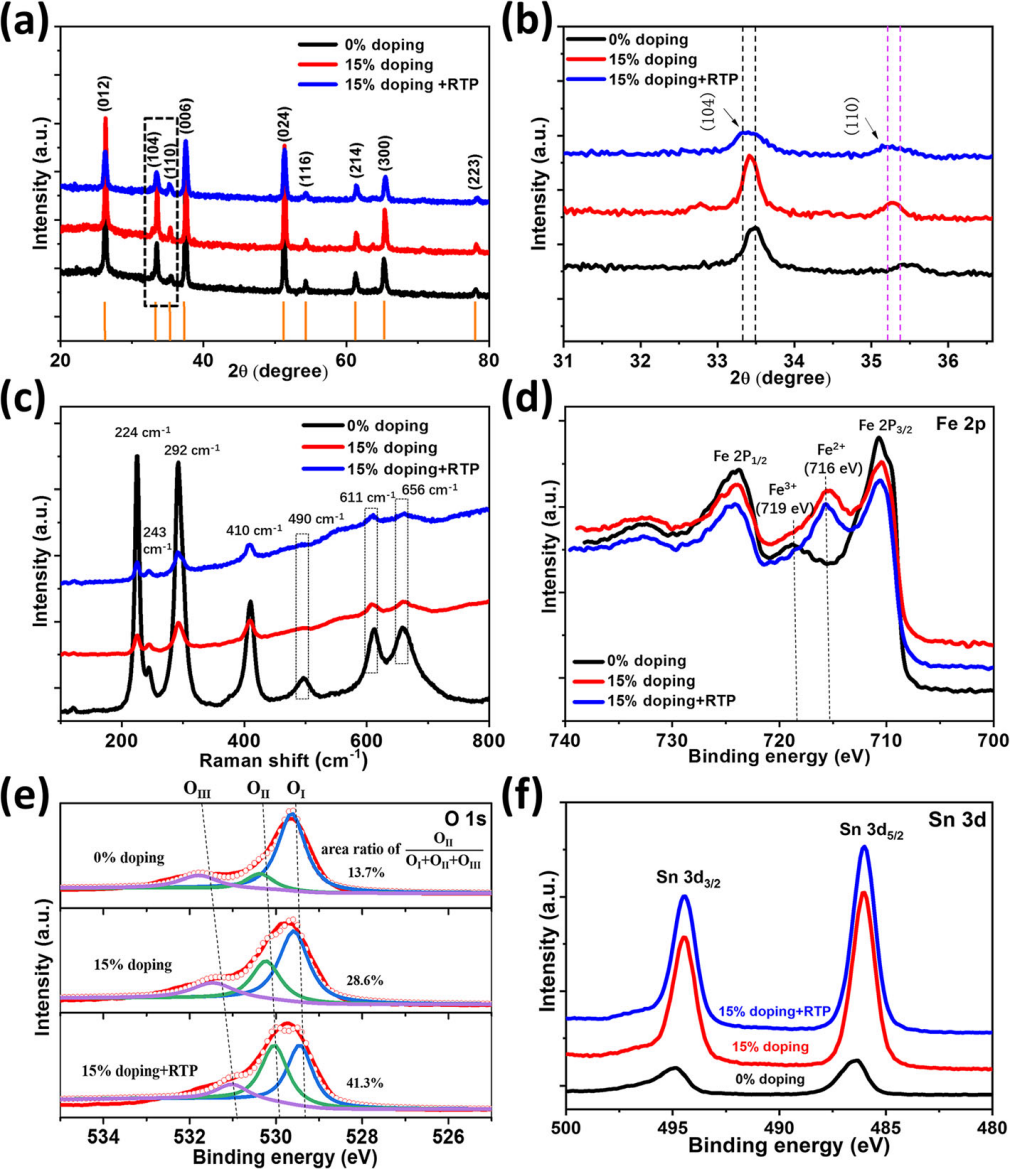
**a** XRD patterns. **b** Locally enlarged image of **a** indicated by the dashed line box. **c** Raman spectra and XPS spectra of **d** Fe 2p. **e** O 1s and **f** Sn 3d in various hematite photoanodes

Figure 2a shows the XRD patterns of the photoanodes with 0% Sn doping, 15% Sn doping, and co-doped by Sn and oxygen vacancy. The characteristic XRD peaks of the three samples indexed to the FTO substrate (JCPDS 46-1088) and hematite (JCPDS 33-0664) show that the α-Fe_2_O_3_ is formed and kept after these treatments (i.e., no great change of crystal structure happened in the process of Sn doping and RTP). What deserves to be mentioned is that the case with 15% Sn doping is not obviously different from the case with 0% Sn doping in the XRD and Raman pattern (Fig. 2a, c). It should be noted that the label of “0% doping” in the manuscript just means no extra element dopant in the precursor solution, but cannot guarantee that the prepared hematite is not doped. Because in the process of post-thermal annealing, the Sn in the FTO substrate can diffuse into the hematite, which is also widely observed by other reports [30]. So the sample labeled as 0% doping in this work in fact is also doped by Sn with a relative low level. With a substantial increase of doping level, a slight shift of the (104) peak in the XRD patterns can be observed from Fig. 2b. These results indicate that a lattice distortion is present after inclusion of high-density impurity atoms. In order to analyze the molecular vibration, Raman spectra are examined. As illustrated in Fig. 2c, obvious Raman peaks indicated that α-Fe_2_O_3_ is synthesized by ultrasonic spraying pyrolysis and post-annealing belongs to trigonal crystal space group symmetry [31]. The photon modes of A_1g_ and E_g_ belong to the symmetric bend of Fe-O and symmetric stretch of O–O along the direction of Fe–O [32]. Raman characteristic peaks located at 243 cm^−1^, 292 cm^−1^, 410 cm^−1^, and 611 cm^−1^ could be attributed to vibrations with symmetry E_g_, while the Raman peaks at 224 cm^−1^ and 490 cm^−1^ are assigned to the A_1g_ modes. In addition, the peak at 656 cm^−1^ represents the grain boundaries in the prepared hematite. With the introduction of Sn, the intensities of these peaks at 224 cm^−1^, 243 cm^−1^, 292 cm^−1^, 410 cm^−1^, and 490 cm^−1^ obviously decrease, implying that Sn doping has a negative effect on the Fe–O bonds and O–O bonds in α-Fe_2_O_3_ [33]. Compared with the 0% doped α-Fe_2_O_3_, the 15% doped samples show no extra Raman peaks. However, the Raman peaks at 611 cm^−1^ and 656 cm^−1^ of the 15% doped α-Fe_2_O_3_ are obviously attenuated, which may be ascribed to the formation of Fe_3_O_4_ [34]. It should be noted that the amount of Fe_3_O_4_ is very small for our RTP-treated α-Fe_2_O_3_, and Fe_3_O_4_ is usually not stable and easy to become Fe_2_O_3_ in air condition. So the formation of Fe_3_O_4_ cannot be directly proved by our XRD pattern. Figure 2d indicates that the signal intensity of Fe^2+^ XPS gets stronger after Sn doping. The existence of oxygen vacancy could be detected by analyzing the O 1s core-level XPS in Fig. 2e. The O 1s peak can be divided into three peaks: O_I_, O_II_, and O_III_ [35]. The lowest binding energy of O 1s (O_I_) that appeared at 529.5 ± 0.1 eV is associated with metal-oxide binding (i.e., the Fe–O bond) [36]. The second O 1s binding energy (O_II_), which is located at 530.2 ± 0.1 eV, corresponds to the oxygen vacancy [37]. The highest binding energy of O 1s (O_III_), which is situated at 531.5 ± 0.1 eV, represents the surface oxygen resulted from the hydrocarbons, surface contamination, and so on [38]. The area ratio of the O_II_ peak to all O 1s is around 13.7% for the 0% doped sample. After introducing 15% doping, the ratio increases to 28.6%. And the ratio for the case combining with Sn doping and RTP is as high as 41.3%. It can be deduced that the combination of Sn doping and RTP with suitable conditions could trigger relatively high density of oxygen vacancy, which is demonstrated to be beneficial for improving the PEC performance. However, the oxygen vacancy with too-high density could become the carrier recombination centers [28]. So the relative density of oxygen vacancy should be carefully controlled. Not only O_III_, but also O_I_ and O_II_ shift in varying degrees. As the Sn dopant is introduced, some Fe atoms are replaced by Sn atoms to produce Sn_Fe_^+^ point defects, and the positive charge Sn_Fe_^+^ would attract the electron cloud of O, so that the O 1s peak has a higher binding energy [39]. Moreover, the further shift of O 1s of the oxygen vacancy and Sn co-doped α-Fe_2_O_3_ indicates that oxygen vacancy has a higher power to attract electron cloud of O [39]. The XPS spectra of Sn in varying photoanodes are shown in Fig. 2f, where the increased peak intensity also proves the rise of Sn doping density.

**Fig. 3:**
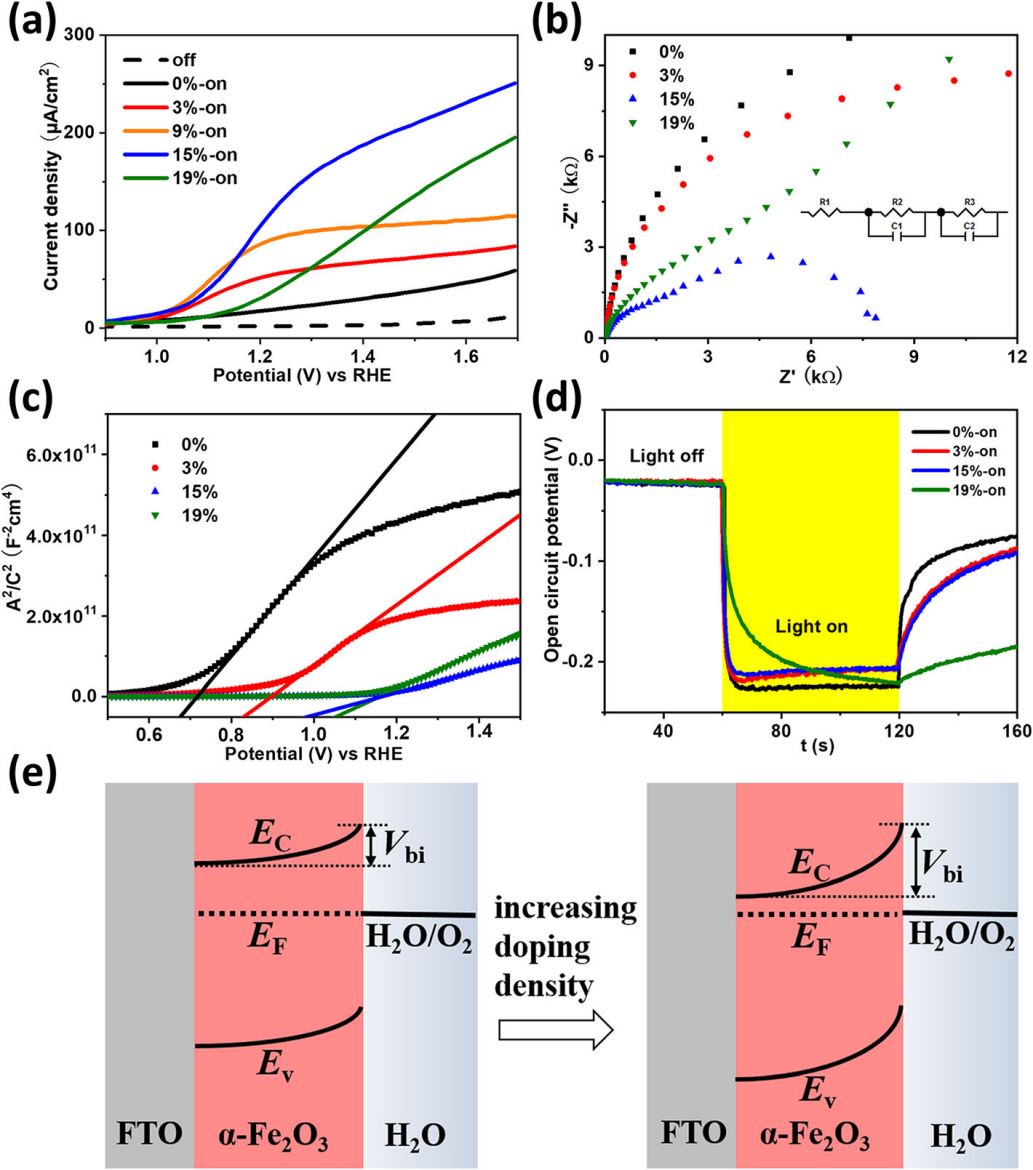
**a** *J-V* curves of the hematite photoanodes with different doping levels in the dark (dashed line) and under one-sun irradiation (solid curves). **b** Photoelectrochemical impedance spectra measured at 1.23 V_RHE_. **c** Mott-Schottky plots. **d** The change of open circuit potential under the light-on/light-off circle. **e** Schematic diagram of the equilibrium state of energy band bending in the dark

Figure 3a compares the *J-V* behaviors of the α-Fe_2_O_3_ photoanodes with different doping levels by adding different amounts of Sn (Cl)_4_ ethanol solution into the Fe (NO)_3_ precursor solution [i.e., 0%, 3%, 9%, 15%, and 19% for the molar ratio (*R*_mol_) of Sn^4+^ to Fe^3+^]. The *J-V* curves for the smaller intervals of doping density are shown in Figure S2. As the doping density continuously increases, the photocurrent (onset potential) first increases (decreases) and then decreases (increases), showing the champion for a suitable doping density (i.e., 10–15%). For the optimized one (i.e., *R*_mol_ = 15%), the *J*_*@*1.23V_ substantially increases to 130 μA/cm^2^ from 10 μA/cm^2^ relative to the 0% doped counterpart, and the onset potential is the smallest (~ 1.0 V_RHE_).

Electrochemical impendence spectrum (EIS) is conducted to reveal the photogenerated carrier dynamics. As shown in Fig. 3b, each Nyquist plot consisted of two semicircles. The radius of the semicircle implies the resistance. The radius of the left (right) semicircle represents the resistance for carriers transferring from hematite inside to the surface states (from surface states into the solution). We used the equivalent circuit inserted in Fig. 3b to fit the EIS. R1, R2, R3, C1, and C2 represent the contact resistance, bulk resistance, transfer resistance, capacitances at the space charge layer of the hematite, and the Helmholtz layer, respectively. Specific fitting values are given in Table S1. When the *R*_mol_ is changed, R1 does not show obvious change, which means that the doping density has an ignorable effect on the contact resistance. R2 is related to the bulk resistance, and it decreases to the minimum (~ 1240 Ω) when *R*_mol_ = 15%. R3 has correlation with surface and solution resistance, and it falls to 5744 Ω when *R*_mol_ = 15%. Comparing the case with *R*_mol_ = 15% to the 0% doped one, R2 shrinks 10 times which means conductivity of α-Fe_2_O_3_ has a great improvement in a large extent. R3 reduces over 13 times, meaning that the introduction of Sn not only reforms the contact surface but also decreases the collision probability of electrons and holes.

The doping density and flat band potential could be estimated from Eqs. (2) and (3) through the Mott-Schottky plots (Fig. 3c).

2 $$ \frac{1}{C^2}=\frac{2}{\varepsilon {\varepsilon}_0{A}^2q{N}_{\mathrm{d}}}\left(E-{E}_{\mathrm{fb}}-\frac{K_{\mathrm{B}}T}{q}\right) $$

3 $$ {N}_{\mathrm{d}}=\left(\frac{2}{\varepsilon {\varepsilon}_0q}\right){\left[\frac{d\left(\raisebox{1ex}{${A}^2$}\!\left/ \!\raisebox{-1ex}{${C}^2$}\right.\right)}{d(E)}\right]}^{-1} $$

where *C* is the capacitance of the space charge region in unit of F, *A* is the projected area of the photoelectrode (~ 0.5 cm^2^), *N*_d_ is the doping density, *q* is electron charge, *ε* is the dielectric constant of hematite (assumed to be 80), *ε*_0_ is the vacuum permittivity, and *E* is the applied potential. For 15% doped hematite, *N*_d_ increases from 1.45 × 10^16^ to 6.37 × 10^16^ cm^−3^ by 4.4 times with respect to the 0% doped hematite. An increase in *N*_d_ from Table S2 confirms the enhancement of conductivity and the reduction of carrier bulk recombination. The flat band potential (*V*_FB_) of the sample with Sn doping gradually shifts to the anodic direction, which confirms that the obtainment of plateau photocurrent needs a large applied potential. To obtain relative information about energy band bending, we tested the open circuit potential (OCP) under the light-on/light-off circle (Fig. 3d). The photo-voltage (i.e., OCP_dark_ − OCP_light_) represents the change of band bending under switching on or off the light because photogenerated carriers in n-type semiconductors will flatten the upward band bending in the dark [40]. The band bending is mainly dependent on the hematite-electrolyte contact situation, like surface recombination and built-in potential (*V*_bi_). In addition, all the Sn-doped samples require longer time to achieve a new equilibrium in the change of light-on state to light-off state. Excessive doping causes the delay of achieving equilibrium state due to the slow photoelectron injection into the surface states. Figure 3e shows the schematic diagram of the equilibrium state of energy band bending in the dark. When the doping density increases, the Femi level of the doped hematite is gradually enhanced. So the degree of the energy band bending at equilibrium state and the *V*_bi_ are larger under a higher doping level. A large *V*_bi_ is beneficial for the separation of the photogenerated carriers, but too-high density doping could result in severe bulk recombination from the crystalline structure defects. The UV-vis absorbance spectra of α-Fe_2_O_3_ film with different doping levels (displayed in Figure S3) indicate that these samples possess nearly the same absorption regardless of the doping density. The absorbance (Abs) spectrum is obtained according to the following formula:

4 $$ \mathrm{Abs}=1-\mathrm{Ref}-\mathrm{Tra} $$

The measured transmittance (Tra) and reflectivity (Ref) spectra are shown in Figure S3(a) and (b). One can see that the intersections for this Abs spectrum are from the Ref spectra, which may be explained by the α-Fe_2_O_3_ photoanodes which have different microscopic morphologies and surface roughnesses. So optical influences of varying doping levels are relatively small.

**Fig. 4:**
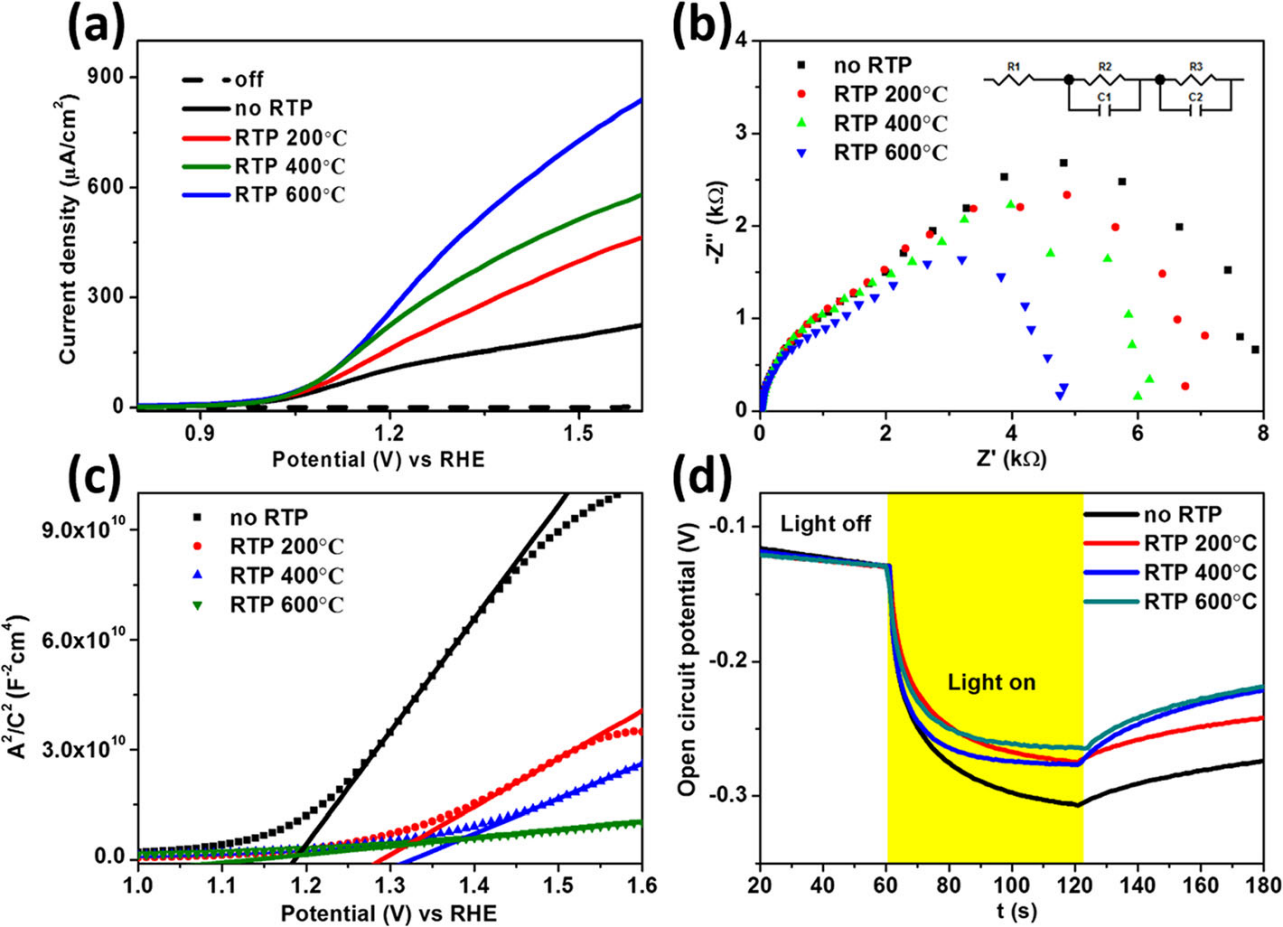
**a** *J-V* curves of the 15% Sn doped hematite photoanode with post-RTP at different temperatures. **b**, **c** The corresponding photoelectrochemical impedance spectra and Mott-Schottky plots, respectively. **d** The change of open circuit potential under the light-on/light-off circle

To further enhance the PEC performance, we carry out the RTP treatment for the 15% doped α-Fe_2_O_3_ film. We focus on temperature (*T*_RTP_) influences. The relative density of oxygen vacancy is decided by RTP conditions. Figure 4a shows the *J-V* curves with different temperatures. It can be seen that the photoanode performance is better for higher *T*_RTP_ in the range of 200–600 °C. Note that further increasing *T*_RTP_ is not implemented successfully due to the cracking of glass substrate. In order to figure out the specific effect of *T*_RTP_, EIS (Fig. 4b) and Mott-Schottky plot (Fig. 4c) are obtained. With an increase of *T*_RTP_, the radii of the two semicircles of EIS curves are obviously reduced, suggesting that the resistances for carrier extraction into the surface states and carrier transfer into the solution are substantially suppressed. The fitted values of resistance and capacitance (using the equivalent circuit inserted in Fig. 3b) are summarized in Table S3, where the R3 relationship is well consistent with the *J-V* behaviors. Meanwhile, the slope of the fitting curve turned down, which means an increase in doping density. Table S4 shows that doping density for the sample with post-RTP at 600 °C is increased to 7.92 × 10^17^ cm^−3^ by ~ 14 times compared to the sample without RTP. The photo-voltage from OCP test (Fig. 4d) shows that a reduction of ~ 20 mV is produced after the RTP implementation, implying that the oxygen vacancy defect is successfully introduced and the surface charges can be more easily transferred into solution. Figure S4(c) shows that the RTP has little effect on the absorbance spectrum of α-Fe_2_O_3_ film, meaning that the significant change from RTP treatment is in the electronic transport properties of the α-Fe_2_O_3_ photoanode. The intersections in Figure S4(c) can also be explained by the same reasons as that in Figure S3(c). The measured Tra and Ref spectra with different RTP temperature are also provided in Figure S4(a) and (b).

**Fig. 5:**
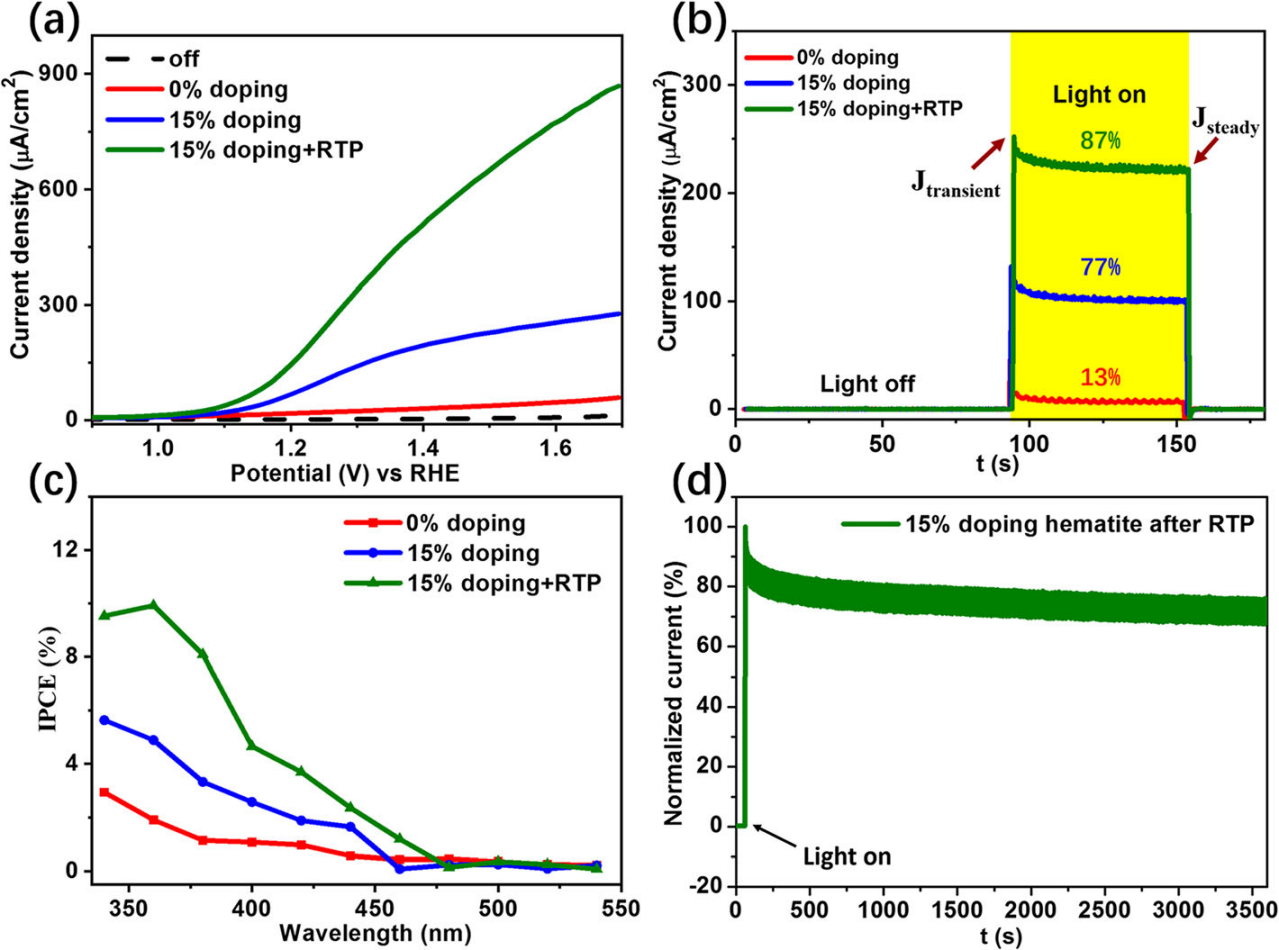
**a** *J-V* curves. **b** Transient current density at 1.23 V_RHE_ under chopped one-sun illumination. The calculated transfer efficiencies are also indicated as a percentage. **c** The IPCE spectrum at 1.23 V_RHE_. **d** The normalized photocurrent at 1.23 V_RHE_ of the Sn-doped hematite photoanode with post-RTP

To intuitively exhibit the enhancement from Sn doping and RTP under the optimized conditions, three samples (i.e., 0% doped, 15% doped, and Sn and oxygen vacancy co-doped α-Fe_2_O_3_ photoanodes) are directly compared. Figure 5 a and b show the process of improvement from the points of *J-V* and *J*_ph@1.23V_ views, respectively. After optimizing the Sn doping, the *J*_ph@1.23V_ from the *J-V* plots is improved by 13 times from 10 to 130 μA/cm^2^. Further introducing RTP improves the *J*_ph@1.23V_ by 25-folds although onset potential is slightly shifted to the right. The transient photocurrent at 1.23 V_RHE_ also shows that the case with co-doping has the best PEC response. The obvious attenuation of transient photocurrent can be explained by the surface recommendation of the photogenerated carriers during the transfer process from photoelectrode surface into electrolyte [41]. Transfer efficiency that shows the decay and overshoot characteristic of surface electron-hole recombination is calculated as the ratio of *J*_transient_ and *J*_steady_ [42, 43]. Here, we define *J*_transient_ as the very beginning photocurrent when the light irradiation is introduced, and *J*_steady_ as the steady photocurrent before turning off the light irradiation. The transfer efficiency is estimated as:

5 $$ \mathrm{transfer}\ \mathrm{efficiency}={J}_{\mathrm{steady}}/{J}_{\mathrm{transient}} $$

The transfer efficiency increases from 13 to 87% for the sample through being co-doped by Sn and oxygen vacancy. It implies that an obvious decrease of the surface recombination rate constant or a substantial increase of the charge transfer rate constant is obtained by the co-doping treatment. Afterwards, *IPCE* at 1.23 V_RHE_ is shown in Fig. 5c. In the *IPCE* measurement, light source is monochromatic. The light intensity of different monochromatic sources for *IPCE* calculation is provided in Figure S5. The overall values of *IPCE* are getting higher with the introduction of co-doping of Sn and oxygen vacancy. Figure 5d shows the normalized *J*_ph@1.23V_, indicating that the PEC response for the Sn and oxygen vacancy co-doped α-Fe_2_O_3_ photoanode is very stable. The obvious decay within the beginning of light illumination is mainly ascribed to the substantial surface carrier recombination (i.e., the non-ideal efficiency of surface charge transfer) [44]. Keeping the illumination for 1 h, the observed photocurrent is around 80% of the original.

Compared with the studies focused on the investigation of extrinsic doping, this work integrates the intrinsic (i.e., oxygen vacancy) and extrinsic (i.e., Sn) dopants. One can see that the Sn doping density should be controlled and moderate, and the RTP conditions have substantial effects on the resultant relative density of oxygen vacancy and the final PEC performances. A combination of Sn doping and introduction of oxygen vacancy can lead to a noticeable improvement relative to these cases with only extrinsic or defect doping, suggesting an effective way to prepare high-performance metal-oxide photoelectrodes.

## Conclusions

The controllable density of Sn doping is introduced into the α-Fe_2_O_3_, which allows the PEC water oxidation performances of the α-Fe_2_O_3_ photoanode to be significantly improved. Our study shows that there is 13-fold enhancement in *J*_ph@1.23V_ for the α-Fe_2_O_3_ photoanode with optimized Sn dopant density, compared to the 0% doped system. With the post-RTP treatment, the PEC performance for the Sn doped hematite can be further enhanced (i.e., by 25-fold enhancement). We ascribe the great improvement to the co-doping of Sn and oxygen vacancy, which can immensely improve the photogenerated carrier separation from the bulk to the surface, as well as the surface charge transfer efficiency. This work provides a universal approach to improve the optoelectronic performance of the metal-oxide semiconductors with poor conductivity and slow kinetics of surface charge transfer.

## Supplementary information

**Additional file 1: Figure S1.** *J-V* curves of hematite photoanodes with different spraying durations in the dark (dashed line) and under one-sun irradiation (solid curves). Note that all the present samples in this figure are treated with the post RTP at 600 °C for 90 s. **Figure S2.** *J-V* curves of hematite photoanodes with different doping levels in the dark (dashed line) and under one-sun irradiation (solid curves). **Figure S3.** The measured transmittance (a), reflectivity (b) and calculated absorbance (c) spectra of hematite photoanodes with different doping levels. **Figure S4.** The measured transmittance (a), reflectivity (b) and calculated absorbance (c) spectra of hematite photoanodes with different RTP temperatures. **Figure S5.** Power density of the single-wavelength light source during the *IPCE* measurement. **Table S1.** The calculated resistance and capacitance values of equivalent circuit according to Figure 3b. **Table S2.** The calculated doping density and flat band potential according to Equations (2 and 3) in Figure 3c. **Table S3.** The calculated resistance and capacitance values of equivalent circuit according to Figure 4b. **Table S4.** The calculated doping density and flat band potential according to Equations (2 and 3) and Figure 4c.

## Data Availability

The relevant data during the experiment are available from the supporting information. The details of the experiment can be obtained from the corresponding author on reasonable request.

## Abbreviations

Abs: Absorbance
EIS: Electrochemical impendence spectrum
IPCE: Incident photon-to-current efficiency
*J*_ph@1.23V_: Photocurrent density at 1.23 V vs. RHE
*J*_steady_: The steady photocurrent
*J*_transient_: The beginning photocurrent
*J-V*: Current density versus potential curves
*N*_d_: Doping density
OCP: Open circuit potential
R1: Contact resistance
R2: Bulk resistance
R3: Transfer resistance
Ref: Reflectivity
*R*_mol_: Molar ratio
RTP: Rapid thermal process
Tra: Transmittance
*T*_RTP_: Temperature of rapid thermal process
*V*_FB_: Flat band potential
