# Correction to: Tin and Oxygen-Vacancy Co-doping into Hematite Photoanode for Improved Photoelectrochemical Performances

**DOI:** 10.1186/s11671-022-03660-0

**Published:** 2022-02-07

**Authors:** Chenhong Xiao, Zhongyuan Zhou, Liujing Li, Shaolong Wu, Xiaofeng Li

**Affiliations:** 1 School of Optoelectronic Science and Engineering & Collaborative Innovation Center of Suzhou Nano Science and Technology, Soochow University, Suzhou, 215006 Jiangsu China; 2 Key Laboratory of Advanced Optical Manufacturing Technologies of Jiangsu Province & Key Laboratory of Modern Optical Technologies of Education Ministry of China, Soochow University, Suzhou, 215006 Jiangsu China

## Correction to: Nanoscale Research Letters (2020) 15:54 10.1186/s11671-020-3287-1

Following publication of the original article [1], it came to the authors’ attention that an incomplete version of affiliation 1 had been provided; ‘Soochow University’ was missing from the affiliation.

The article has now been updated with the corrected affiliation.

## References

[1] Xiao C, Zhou Z, Li L (2020). Tin and oxygen-vacancy co-doping into hematite photoanode for improved photoelectrochemical performances. Nanoscale Res Lett.

