# Supplementary material for: Tin and Oxygen-Vacancy Co-doping into Hematite Photoanode for Improved Photoelectrochemical Performances
Source: Nanoscale Res Lett. 2020 Mar 4;15:54. doi: 10.1186/s11671-020-3287-1 (PMC7056762; doi:10.1186/s11671-020-3287-1)
Supplement: Supplementary file 1 — Additional file 1: Figure S1. J-V curves of hematite photoanodes with different spraying durations in the dark (dashed line) and under one-sun irradiation (solid curves). Note that all the present samples in this figure are treated with the post RTP at 600 °C for 90 s. Figure S2. J-V curves of hematite photoanodes with different doping levels in the dark (dashed line) and under one-sun irradiation (solid curves). Figure S3. The measured transmittance (a), reflectivity (b) and calculated absorbance (c) spectra of hematite photoanodes with different doping levels. Figure S4. The measured transmittance (a), reflectivity (b) and calculated absorbance (c) spectra of hematite photoanodes with different RTP temperatures. Figure S5. Power density of the single-wavelength light source during the IPCE measurement. Table S1. The calculated resistance and capacitance values of equivalent circuit according to Figure 3b. Table S2. The calculated doping density and flat band potential according to Equations (2 and 3) in Figure 3c. Table S3. The calculated resistance and capacitance values of equivalent circuit according to Figure 4b. Table S4. The calculated doping density and flat band potential according to Equations (2 and 3) and Figure 4c. [file 11671_2020_3287_MOESM1_ESM.docx]

Supporting information

**Tin and Oxygen-Vacancy Co-doping into Hematite Photoanode for Improved Photoelectrochemical Performances**

Chenhong Xiao^1,2^, Zhongyuan Zhou^1,2^, Liujing Li^1,2^, Shaolong Wu^1,2,*^ and Xiaofeng Li^1,2,*^

*^1^School of Optoelectronic Science and Engineering & Collaborative Innovation Center of Suzhou Nano Science and Technology, Suzhou 215006, Jiangsu, China*

*^2^Key Lab of Advanced Optical Manufacturing Technologies of Jiangsu Province & Key Lab of Modern Optical Technologies of Education Ministry of China, Soochow University, Suzhou 215006, Jiangsu, China*

*^*^E-mail: shaolong_wu@suda.edu.cn; xfli@suda.edu.cn*

**
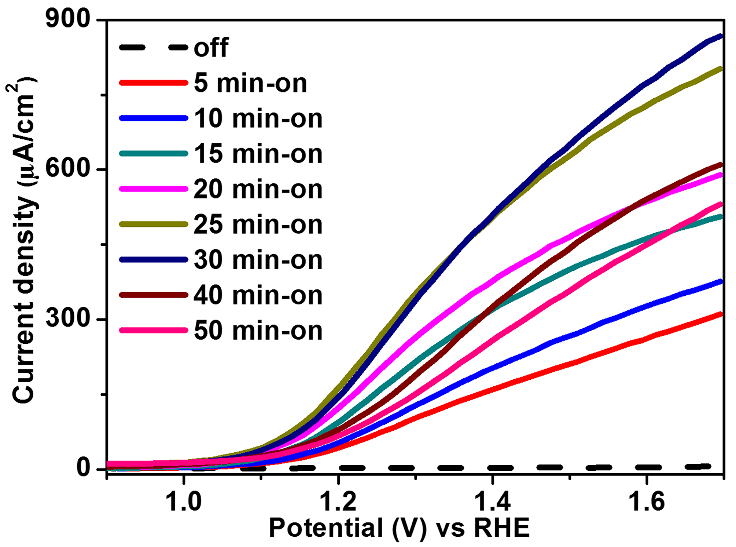
**

**Figure S1.** *J-V* curves of hematite photoanodes with different spraying durations in the dark (dashed line) and under one-sun irradiation (solid curves). Note that all the present samples in this figure are treated with the post RTP at 600 °C for 90 s.

**
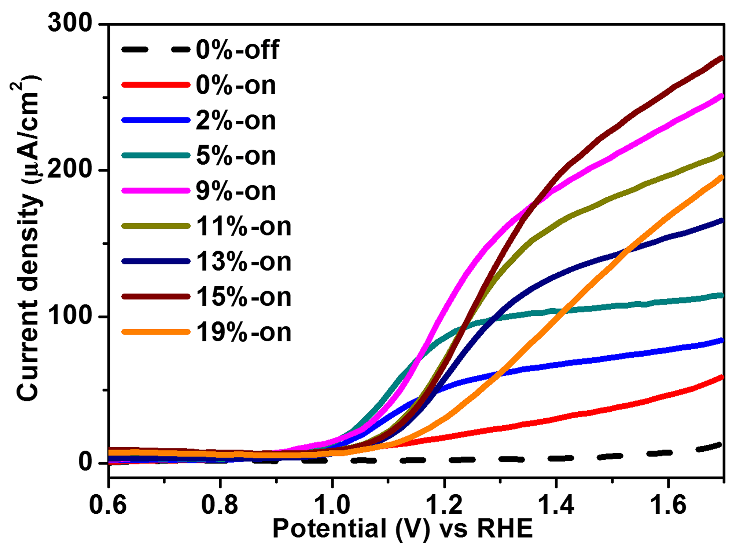
**

**Figure S2.** *J-V* curves of hematite photoanodes with different doping levels in the dark (dashed line) and under one-sun irradiation (solid curves).

**
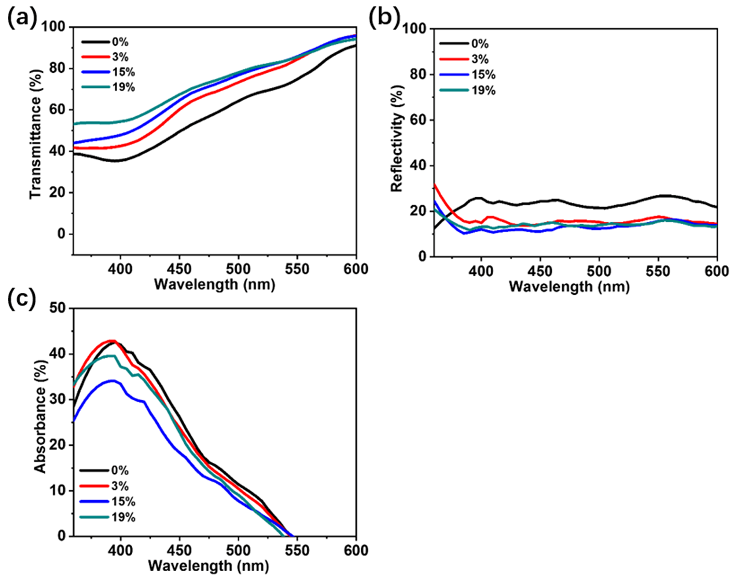
**

**Figure S3.** The measured transmittance (a), reflectivity (b) and calculated absorbance (c) spectra of hematite photoanodes with different doping levels.

**
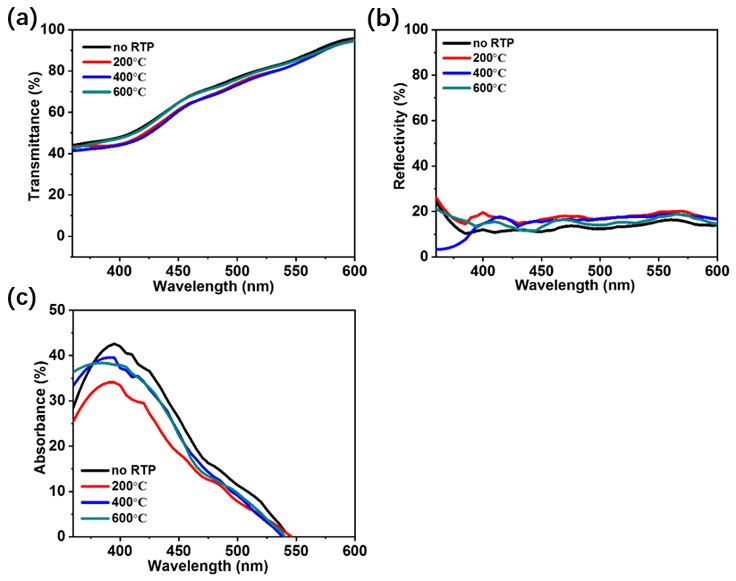
**

**Figure S4.** The measured transmittance (a), reflectivity (b) and calculated absorbance (c) spectra of hematite photoanodes with different RTP temperatures.

*
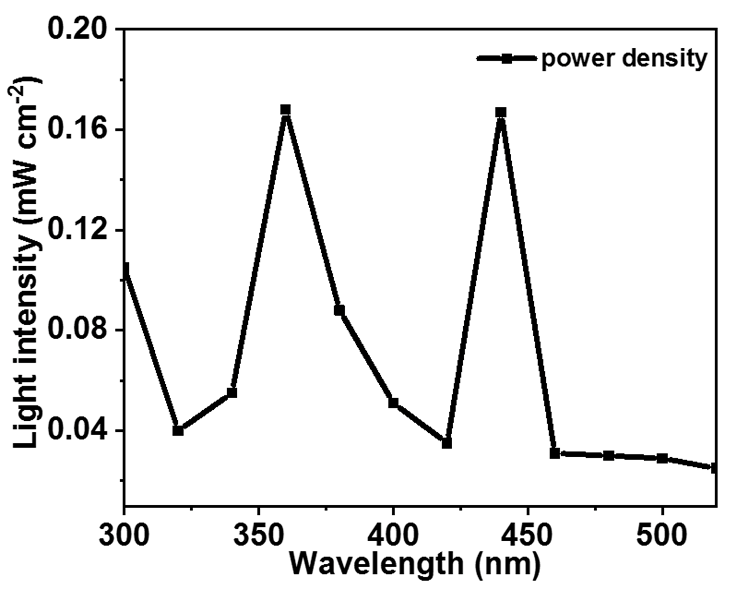
*

**Figure S5.** Power density of the single-wavelength light source during the *IPCE* measurement.

**Table S1.** The calculated resistance and capacitance values of equivalent circuit according to Figure 3(b).

| **Sample** | **R1 (Ω)** | **R2 (Ω)** | **R3 (Ω)** | **C1 (F)** | **C2 (F)** |
| --- | --- | --- | --- | --- | --- |
| 0% | 33.2 | 15470 | 78414 | 3.7**×**10^-7^ | 2.6**×**10^-6^ |
| 3% | 20.1 | 14231 | 45760 | 3.0**×**10^-7^ | 3.8**×**10^-6^ |
| 15% | 14.9 | 1240 | 5744 | 1.4**×**10^-6^ | 1.0**×**10^-5^ |
| 19% | 24.6 | 3547 | 28369 | 2.4**×**10^-7^ | 2.0**×**10^-6^ |

**Table S2.** The calculated doping density and flat band potential according to Equations (2 and 3) in Figure 3(c).

| **Sample^a^** | ***N*_d_ (×10^16^ cm^-3^)^b^** | ***E*_FB_ (V)^c^** |
| --- | --- | --- |
| 0% | 1.45 | 0.7 |
| 3% | 2.36 | 0.8 |
| 15% | 6.37 | 1.0 |
| 19% | 3.77 | 1.1 |

^a^Unless specially mentioned, all the sample are sprayed for 30 minutes and then pre-annealed at 700 °C for 2h without post-rapid thermal process; the difference is the Sn-doped density.

^b^*N*_d_: Doping density

^c^*E*_FB_: Flat band potential vs RHE

**Table S3.** The calculated resistance and capacitance values of equivalent circuit according to Figure 4(b).

| **Sample** | **R1 (Ω)** | **R2 (Ω)** | **R3 (Ω)** | **C1 (F)** | **C2 (F)** |
| --- | --- | --- | --- | --- | --- |
| No RTP | 14.9 | 1240 | 5744 | 1.4**×**10^-6^ | 1.0**×**10^-5^ |
| 200 °C | 14.9 | 1157 | 5146 | 1.5**×**10^-6^ | 9.0**×**10^-6^ |
| 400 °C | 16.0 | 1164 | 4476 | 1.3**×**10^-6^ | 8.3**×**10^-6^ |
| 600 °C | 19.0 | 1024 | 3448 | 2.0**×**10^-6^ | 1.3**×**10^-5^ |

**Table S4.** The calculated doping density and flat band potential according to Equations (2 and 3) and Figure 4(c).

| **Sample^1^** | ***N*_d_ (×10^16^ cm^-3^)** | ***E*_FB_ (V)** |
| --- | --- | --- |
| No RTP | 5.7 | 1.2 |
| 200 °C | 13.5 | 1.3 |
| 400 °C | 18.6 | 1.3 |
| 600 °C | 79.2 | 1.1 |

^1^Unless specially mentioned, all the sample were sprayed for 30 minutes with 15% Sn doping and then pre-annealed at 700 °C and post-RTP; only difference between these samples is the temperature of RTP.
